# Noninvasive Assessment of Testosterone Levels and Male Sexual Behavior in Sambar Deer (*Rusa unicolor*) as a Critical Step Toward Conservation in Captivity

**DOI:** 10.1155/vmi/8090018

**Published:** 2025-12-28

**Authors:** Gholib Gholib, Dara Cut Rinjani, Muslim Akmal, Sri Wahyuni, Taufiq Purna Nugraha

**Affiliations:** ^1^ Physiology Laboratory, Faculty of Veterinary Medicine, Universitas Syiah Kuala, Banda Aceh, 23111, Aceh, Indonesia, unsyiah.ac.id; ^2^ Research Center for Applied Zoology, National Research and Innovation Agency, Bogor, Indonesia, brin.go.id; ^3^ Study Program of Veterinary Medicine, Faculty of Veterinary Medicine, Universitas Syiah Kuala, Banda Aceh, 23111, Aceh, Indonesia, unsyiah.ac.id; ^4^ Histology Laboratory, Faculty of Veterinary Medicine, Universitas Syiah Kuala, Banda Aceh, 23111, Aceh, Indonesia, unsyiah.ac.id; ^5^ Anatomy Laboratory, Faculty of Veterinary Medicine, Universitas Syiah Kuala, Banda Aceh, 23111, Aceh, Indonesia, unsyiah.ac.id

**Keywords:** antler cycle, mating behavior, *Rusa unicolor*, sambar deer, testosterone

## Abstract

The role of testosterone in regulating the antler cycle and sexual behavior in temperate cervids is well documented. However, studies on tropical cervids, such as the sambar deer (*Rusa unicolor*), remain limited. This study aimed to measure testosterone levels and investigate their relationship with sexual behavior across different antler stages using a noninvasive approach. Eight sambar deer stags aged 3–5 years, housed in the Taman Rusa Zoo, Aceh, Indonesia, were observed. Fecal samples were collected 1–3 times per week from each individual, along with the behavior observation and antler stage assessments. Sexual behaviors were carried out using focal animal sampling and recorded using the all‐occurrence sampling method. Levels of testosterone in feces were measured using the enzyme‐linked immunosorbent assay technique. Data were analyzed using a linear mixed‐effects model with antler stage as a fixed effect and individual identity as a random factor. Spearman’s rank correlation test was used to examine the relationship between testosterone levels and sexual behavior. The results revealed a clear association between testosterone levels, antler stages, and male sexual behaviors. Levels of testosterone varied significantly among antler stages, with higher levels found during the hard and velvet stages compared to the casting stage (*p* < 0.01). Moreover, a strong positive correlation was found between testosterone levels and male sexual behavior during the hard antler stage (rs = 0.763, *p* < 0.01). Despite high testosterone levels during the velvet stage, male sexual behaviors, such as anogenital sniffing, anogenital licking, and flehmen, were significantly lower compared to the hard antler stage. These results suggest that testosterone may play additional roles beyond those related to male competition or mating during the velvet stage, which warrants further investigation. Understanding the dynamics of testosterone and male sexual behavior is crucial for the effective management and conservation of sambar deer populations in captivity.

## 1. Introduction

Indonesia boasts a rich faunal diversity, including the sambar deer (*Rusa unicolor*). This species, classified under Cervidae, comprises two subspecies: *Rusa unicolor brookei* on Kalimantan Island and *Rusa unicolor equinus* on Sumatera Island, Indonesia [1]. However, illegal hunting and habitat loss have led to a decline in the sambar deer population. Consequently, the International Union for Conservation of Nature (IUCN) has listed the species as vulnerable since 2015 [2]. Captive breeding programs for sambar deer are therefore crucial, not only for biodiversity conservation but also for providing an alternative source of meat.

Successful captive breeding requires a thorough understanding of the reproductive physiology and behavior. While considerable study has focused on the female reproductive cycle [3], the male contribution remains comparatively understudied. In sambar deer, the antler cycle comprising hard antlers, shedding, and velvet antlers is closely associated with fluctuations in testosterone [4]. In white‐tailed deer (*Odocoileus virginianus*), testosterone stimulates antler growth, muscle development, and scent‐marking activities [5], and it is significantly associated with sperm quality [6, 7]. In Iberian red deer (*Cervus elaphus hispanicus*), testosterone levels were low during early antler growth (spring), rising sharply in late summer, peaking just before the rut in autumn [8].

In cervids, testosterone plays a central role in regulating antler growth, spermatogenesis, and sexual behavior. In temperate species, such as the red deer (*Cervus elaphus*), white‐tailed deer, and elk (*Cervus canadensis*), processes are highly seasonal and driven primarily by photoperiod [6, 8–10]. Rising testosterone levels in late summer stimulate the transition to hard antlers and peak reproductive activity during the rut, followed by testicular regression and antler casting as testosterone declines after the breeding season [8]. In contrast, tropical cervids, such as sambar deer, exhibit less synchronized cycles, with reproductive activity and antler phases occurring year‐round [4, 11]. While the role of testosterone remains critical across both groups, studies on male hormonal reproduction and antler cycles in tropical cervids remain limited, leaving significant gaps in understanding their reproductive strategies in nonseasonal environments. Thus, assessing testosterone levels and observing sexual behavior are crucial for captive breeding success.

In recent years, a noninvasive method for assessing testosterone levels from fecal samples has become invaluable, especially for stress‐prone species, such as sambar deer. This approach boasts advantages over invasive techniques, including easier sample collection, less disturbance to animals, and less stress during handling [12]. Additionally, fecal samples contain metabolized steroid hormones, such as testosterone, making them suitable for hormone analysis. Thus, this study aimed to noninvasively measure testosterone levels in feces and investigate their relationship with sexual behavior across various antler stages.

## 2. Materials and Methods

### 2.1. Study Site and Animals

The study was conducted at the Taman Rusa Zoo, located in Lamtanjong, Aceh Besar, Aceh, Indonesia (5°27′02.3″N, 95°22′04.8″E), over three months. Eight stags (adult male *Rusa unicolor*) aged between 3 and 5 years were included in the study. The animals were housed in a managed facility. The classification of adult stags was determined primarily from zoo records and further verified using biological indicators of sexual maturity, including antler length greater than 30 cm and evidence of previous antler casting and subsequent regrowth, as previously described for sambar deer [13]. Due to the limited study duration, a full antler cycle could not be observed in all individuals, resulting in variation in antler development stages among individuals. During the observation period, the antler development stages of eight adult sambar stags were observed. Each individual was classified into a specific antler phase based on visual assessment of antler morphology. The distribution of individuals across antler phases observed during the study period is summarized in Table 1.

**Table 1:** Antler phase of sambar stags during the observation period.

Stag ID	Antler phase description	Classification
RS01	Fully mineralized antlers, no velvet present	Hard antler
RS02	Fully mineralized antlers, no velvet present	Hard antler
RS03	Fully mineralized antlers, no velvet present	Hard antler
RS04	Soft antlers covered with velvet, active growth phase	Velvet
RS05	Soft antlers covered with velvet, active growth phase	Velvet
RS06	Transitioning from the hard antler phase to casting and early velvet	Hard–casting–velvet
RS07	Transitioning from the hard antler phase to casting and early velvet	Hard–casting–velvet
RS08	Recently cast antlers; early velvet regrowth visible	Casting–early velvet

These sambar deer stags were housed in enclosures covering an area of 429 m^2^. The enclosures were facilitated with two shaded areas (measuring 4 and 2.5 m^2^, respectively), two feeding troughs, a watering facility, and natural enrichments, such as trees. Additionally, a separate isolation enclosure sized 33 m^2^ was also available when needed. Deer were fed twice daily, and water access was provided ad libitum. The diet consisted of grass, foliage, fruit peels, and concentrated feed supplements once a week. Fecal samples were collected in the morning between 08.00 and 10.00 a.m. at a frequency of 1–3 samplings per week from each individual during mating behavior observations.

### 2.2. Male Sexual Behavior and Antler Stage Observations

Male sexual activity and antler growth stages were observed using the focal animal sampling method from 8.00 a.m. to 5.00 p.m. three times a week [14]. Male sexual behaviors were quantified using the all‐occurrence sampling method [15]. Every instance of predefined behaviors (e.g., anogenital sniffing, anogenital licking, and flehmen) was continuously recorded during observation sessions, allowing determination of both frequency and duration for each behavior expressed by individual stags. These behaviors were classified into three categories: (a) precopulatory behaviors, (b) copulatory behaviors, and (c) postcopulatory behaviors. An ethogram delineating male sexual behavior was crafted, adopted from previous studies, as outlined in Table 2 [16, 17]. In conjunction with observing sexual behavior, we also monitored and documented the progression of antler stages in each male deer.

**Table 2:** Ethogram of male sexual behavior and specific events in each stage of the sambar deer stag.

Stage of male sexual behavior	Specific events	Code	Description
Precopulatory behavior	Male approach	ma	The male moves closer within 1–2 m of a female and orients his head toward her
	Male follows	mf	The male closely follows a female for > 10 s, maintaining proximity (< 1 m) while tracking her movements
	Courtship Includes:		
	‐ Anogenital sniffing	as	The male presses his nose into the female’s anogenital region and sniffs for ≥ 2 s to detect olfactory cues
	‐ Anogenital licking	al	The male uses his tongue to make repeated contact with the female’s genital area, typically sustained for 2–5 s
	‐ Flehmen	fl	The male lifts his head, curls his upper lip, and exposes incisors after sniffing urine or the anogenital region, lasting 1–3 s
	Smelling of female urine	sfu	The male lowers his muzzle to fresh urine patches left by a female and inhales for > 2 s
	Erection	er	The male’s penis becomes enlarged, firm, and erect, generally occurring immediately before mounting

Copulatory behavior	Mounting	mo	The male positions his thorax on the female’s rump, forelimbs resting on her back, hindlimbs on the ground; mounting lasts ≥ 3 s
	Copulation	co	The male inserts his erect penis into the female’s reproductive tract, accompanied by pelvic thrusts, typically for 5–15 s

Postcopulatory behavior	Dismount	ds	The male dismounts after copulation and remains motionless behind the female for 2–5 s
	Grooming	gr	The male licks the female’s body (neck, flank, or rump) for ≥ 5 s, often occurring immediately after copulation

### 2.3. Fecal Sample Collection

Before sample collection, a preliminary assessment was conducted to determine the average weight of individual fecal pellets, which ranged between 0.7 and 0.8 g per pellet. Based on this measurement, approximately 30 pellets were collected per sample collection time to obtain ∼20 g of fecal material. Fresh fecal samples were collected immediately after defecation by manually picking pellets directly from the ground using a clean stick. To ensure sample quality, only pellets free from soil, small pebbles, grass residues, and urine contamination were selected. Samples were put in vials per collection time and temporarily stored in a cool box during fieldwork, and then stored at −20°C for approximately one month in the laboratory until being processed.

### 2.4. Fecal Sample Preparation and Extraction

Before extraction, samples were thawed overnight at 4°C. The fecal extraction procedure was adapted from the method described by Gholib et al. in a previous study on sambar deer [12]. About 0.5–0.6 g of each fecal sample was placed into a 15‐mL centrifuge tube containing 4.5 mL of 80% ethanol. The mixture was then vortexed using a multivortexer at 1000 rpm for 15 min (Brand, USA). Subsequently, the fecal solution was then centrifuged at 3000 rpm for 10 min. A volume of 1 mL of the supernatant was transferred to a 1.5‐mL microtube and incubated at 40°C to evaporate the ethanol. After complete evaporation, the dried fecal extract was reconstituted with 1 mL of assay buffer and later was stored at −20°C before testosterone measurement.

### 2.5. Assay Validation and Testosterone Level Measurements

Levels of testosterone were quantified using a commercial testosterone enzyme‐linked immunosorbent assay (ELISA) kit (Cat. EIA‐1559, DRG Instruments GmbH, Germany), which has been applied to various animal samples [18, 19]. To ensure the suitability of this assay for fecal hormone measurements in sambar deer, additional validation procedures were performed before sample analysis. Analytical validation of the testosterone ELISA kit was performed to assess parallelism, dose–response characteristics, accuracy, and reproducibility. Parallelism was evaluated by preparing serial dilutions of sambar stag fecal extract samples ranging from 1:2 to 1:32, which were assayed together with the testosterone standard. The slopes of the expected dose versus percent bound from the diluted sambar stag samples were compared with the slope of the testosterone standard curve to confirm parallel displacement. Accuracy was determined by spiking known quantities of testosterone into the testosterone standard and calculating the percentage recovery. Reproducibility was assessed by analyzing low‐quality and high‐quality controls within a single assay to obtain intra‐assay coefficients of variation (CV), and across multiple assays to determine interassay CV. Cross‐reactivity profiles and the limit of detection followed the specifications provided in the manufacturer’s manual, with reported cross‐reactivity of 100% with testosterone, 12.9% with dihydrotestosterone (DHT), 3.3% with 11β‐hydroxytestosterone, 3.3% with 19‐nortestosterone, and < 1% with other hormones. The limit of detection of the assay was 0.15 ng/mL. Biological validation was additionally performed by comparing testosterone concentrations among adult males, juvenile males, and adult females to confirm physiological differentiation. The procedure of testosterone measurements was conducted as described in the manual of the ELISA kit.

### 2.6. Data Analysis

Before data analysis, normality and homogeneity of data were evaluated using the Shapiro–Wilk test and Levene’s homogeneity test. For the analytical validation of the assay, parallelism between the serial dilutions of fecal extract from a sambar stag and the standard curve was assessed by comparing the equality of slopes using logarithmic regression analysis. For the biological validation, testosterone data from adult males, juveniles, and adult females did not meet the assumptions of normality (*p* < 0.05). Therefore, group differences were analyzed using the Kruskal–Wallis test followed by the Mann–Whitney U post hoc comparisons. Testosterone concentrations were analyzed using a linear mixed‐effects model, with antler stage included as a fixed effect and individual identity as a random effect to account for repeated measurements. Testosterone values were log‐transformed before analysis to improve normality and stabilize variance. Moreover, Spearman’s rank correlation test was employed to assess the relationship between testosterone levels and mating behaviors. In analyzing the correlation between fecal testosterone and behavioral variables, a time lag of approximately 1–2 days was considered to account for the delay between hormone secretion into circulation and its appearance in feces [20, 21]. All statistical analyses were conducted at a significance level of *α* = 0.05. Data were reported as means and standard deviations (SD) and analyzed using IBM SPSS Statistics Version 25. Graphical representations were produced using SigmaPlot Version 12.5.

## 3. Results

### 3.1. Validation of the Testosterone ELISA Kit

Serial dilutions of fecal extracts from a sambar stag produced displacement curves that were parallel to the testosterone standard curve (Figure 1(a)). As expected, measured testosterone concentrations declined progressively with increasing dilution. From the dose–response analysis, the slope of the testosterone standard curve was −20.626, while the diluted fecal extract had a slope of −25.150. The test for equality of slopes showed no significant difference between the curves (*p* > 0.05), confirming parallelism and demonstrating that the assay reliably detects testosterone across the dilution range. The results of the parallelism test, dose–response characteristics, accuracy, and assay reproducibility (intra‐ and interassay CVs) are summarized in Table 3.

Figure 1(a) Parallelism test curves for serial dilutions of sambar stag fecal extracts (white circles) and testosterone standards (black circles). Fecal extracts were diluted 1:2 to 1:32 in assay buffer and measured against serial testosterone standards ranging from 0.2 to 16 ng/mL. The curves for fecal extracts and standards showed nearly identical coefficients of determination (*R* ^2^ = 0.978 and 0.982, respectively), indicating good parallelism. (b) Box plot showing fecal testosterone levels in adult males, juvenile males, and adult females of sambar deer. Asterisks (^∗∗^) indicate significant differences between groups (*p* < 0.01).

**Figure:**
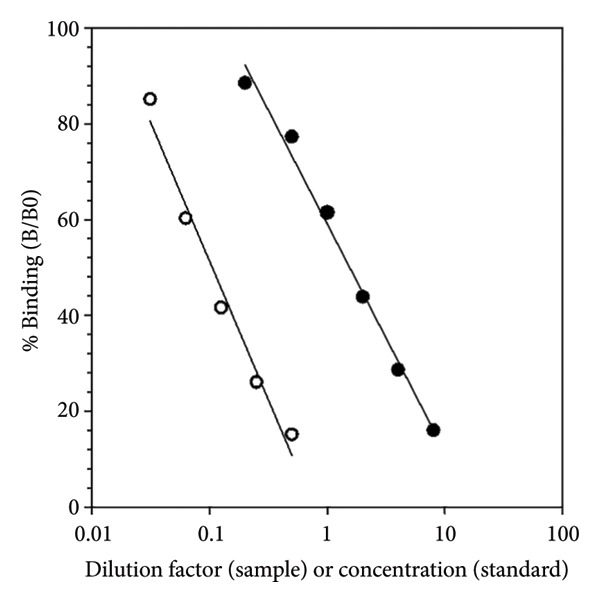
(a)

**Figure:**
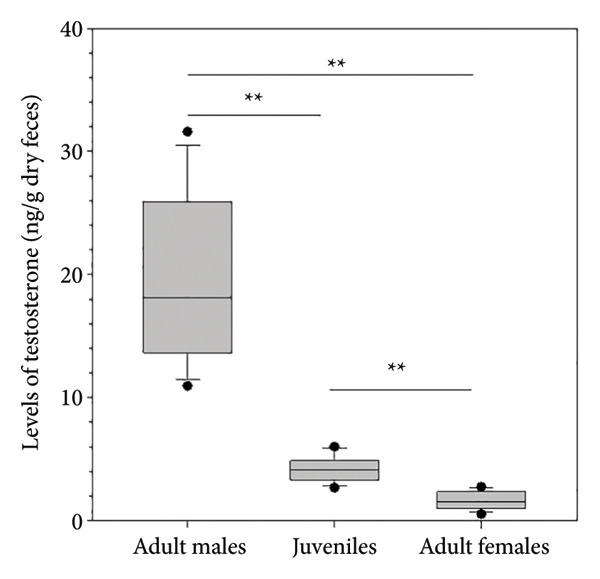
(b)

**Table 3:** Results of the analytical validation of a commercial testosterone ELISA kit (Cat. EIA‐1559, DRG Instruments GmbH, Germany) for testosterone measurement in the feces of sambar deer.

Measured parameters	Results
Parallelism	Parallel, *z* = 1.681, *p* = 0.093
Dose–response curve	
Standard	*Y* = −20.626 Ln (*x*) + 59.025, *R* ^2^ = 0.982, *p* = 0.001
Sample	*Y* = −25.150 Ln (*x*) − 6.661, *R* ^2^ = 0.978, *p* = 0.001
Accuracy ± SD (%)	96.32 ± 5.16
Coefficient of variation (CV) of intra‐assay (%)	
Low‐quality control (*N* = 6)	6.23
High‐quality control (*N* = 6)	8.11
Coefficient of variation (CV) of interassay (%)	
Low‐quality control (*N* = 3)	9.52
High‐quality control (*N* = 3)	8.32

For biological validation, testosterone levels differed markedly among adult males, juvenile males, and adult females (*p* < 0.01, Figure 1(b)). Testosterone concentrations were significantly higher in adult males compared to both juvenile males and adult females (*p* < 0.01). Additionally, juvenile males exhibited significantly higher testosterone levels than adult females (*p* < 0.01).

### 3.2. Testosterone Levels of Sambar Deer in Different Antler Stages

Before casting, sambar deer stags in the hard antler stage frequently exhibited antler‐rubbing behavior against trees or enclosure structures. Typically, one antler was shed first, followed by the second within 1–2 days. Antler regrowth commenced 2–3 weeks after casting. During this stage, antlers initially appeared as small buds, gradually developing into a Y‐shape, and eventually forming three distinct branches. This growth was accompanied by wound healing processes at the antler base, characterized by active cellular proliferation and tissue regeneration (Figure 2(a)). These changes in antler stages were accompanied by fluctuations in testosterone levels. Specifically, testosterone levels appeared to peak during the hard antler stage, declined during casting, and then gradually increased during the velvet stage (Figure 2(b)).

Figure 2(a) Antler stage and (b) testosterone levels in a sambar deer stag: hard antler (a‐1), casting (a2‐a3), and velvet phases (a4).

**Figure:**
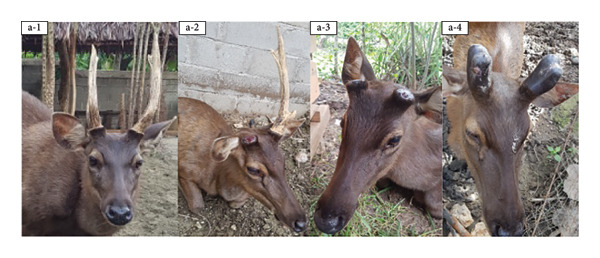
(a)

**Figure:**
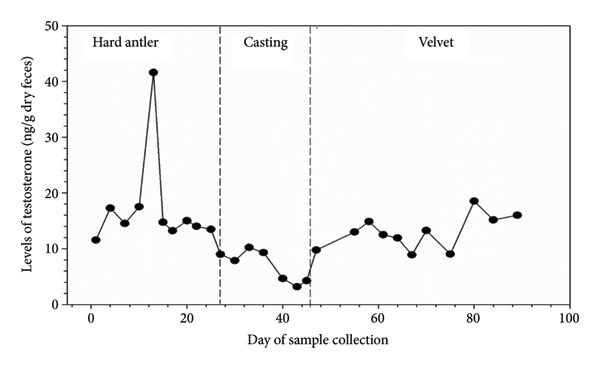
(b)

**Figure 3:**
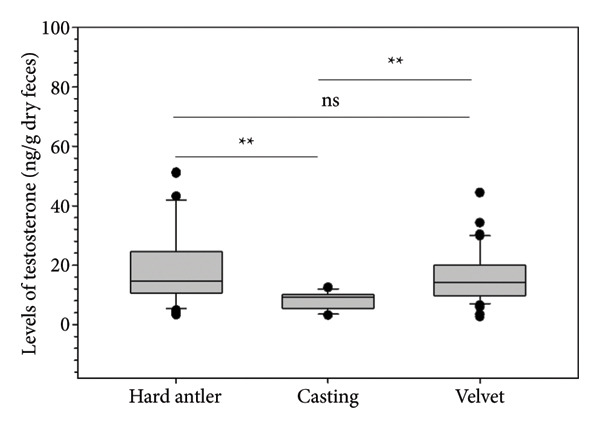
Box plot showing fecal testosterone levels in sambar deer across different antler stages at Taman Rusa Lamtanjong, Aceh, Indonesia. Asterisks (^∗∗^) indicate significant differences (*p* < 0.01), and “ns” indicates nonsignificant differences (*p* > 0.05).

Linear mixed modeling of log‐transformed testosterone levels revealed a significant effect of antler stage on testosterone levels (*p* < 0.01, Figure 3). Post hoc comparisons showed no difference between the hard antler and velvet phases (*p* > 0.05), whereas testosterone levels during the casting phase were significantly lower than both hard antler and velvet stages (*p* < 0.01). Individual identity was retained as a random effect and accounted for the repeated sampling structure.

### 3.3. Male Sexual Behavior of Sambar Deer

**Figure 5:**
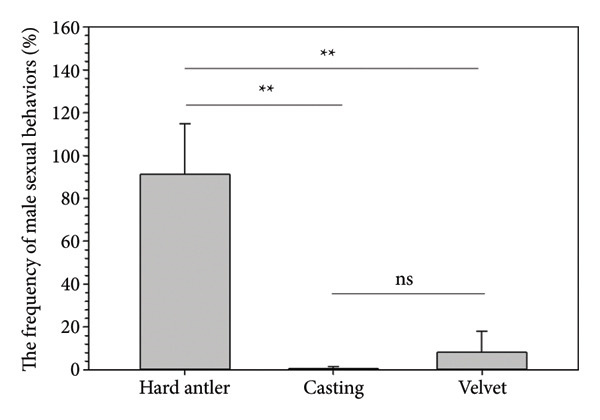
The frequency of sexual behaviors of sambar deer in different antlers. Asterisks (^∗∗^) indicate significant differences (*p* < 0.01), and “ns” indicates nonsignificant differences (*p* > 0.05).

Observations of sexual behavior revealed a total of 11 specific behaviors exhibited by the sambar deer stag (Figure 4, Table 4). Among the observed sexual behaviors, 91.40% of male sexual behaviors occurred during the hard antler, 8.07% occurred during the growing antler (velvet), while only 0.54% of sexual behaviors were observed during the shedding antler (casting). Statistical analysis indicated a significant difference in frequency of sexual behaviors across different antler stages (*p* < 0.01, Figure 5). Furthermore, statistical analysis indicated a highly positive correlation between the number of sexual behaviors and testosterone levels (rs = 0.763, *p* < 0.01) shown during the hard antler (Figure 6(a)). However, no significant correlation was observed between the number of sexual behaviors and testosterone levels during the growth of antler or velvet (Figure 6(b)) or the casting/shedding of antler (Figure 6(c)).

Figure 4Sequences of male sexual behavior in sambar deer: precopulatory behavior (a–g), copulatory behaviors (h–j), and postcopulatory behavior (k–l). (a) male approach, (b) male follow, (c) anogenital sniffing, (d) anogenital licking, (e) flehmen, (f) smelling female’s urine, (g) erection, (h, i) mounting, (j) copulation, (k) dismount, and (l) grooming.

**Figure:**
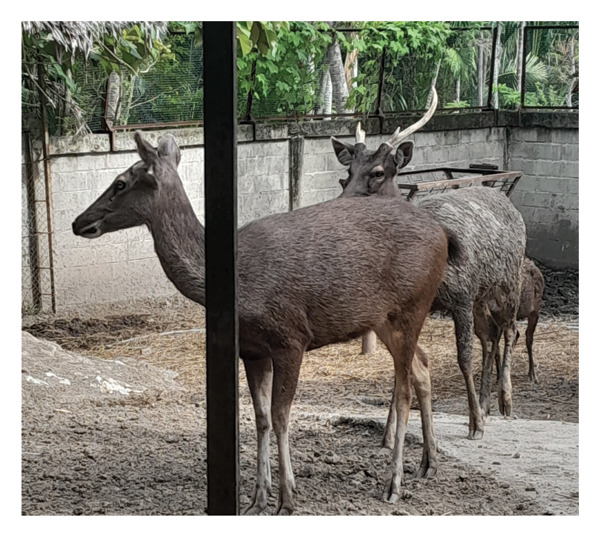
(a)

**Figure:**
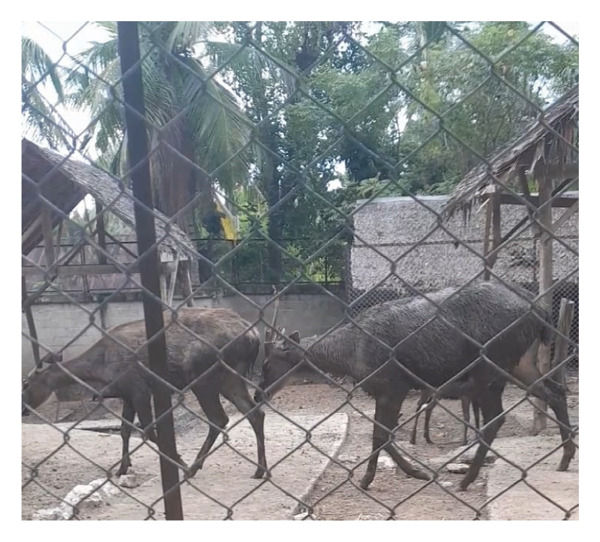
(b)

**Figure:**
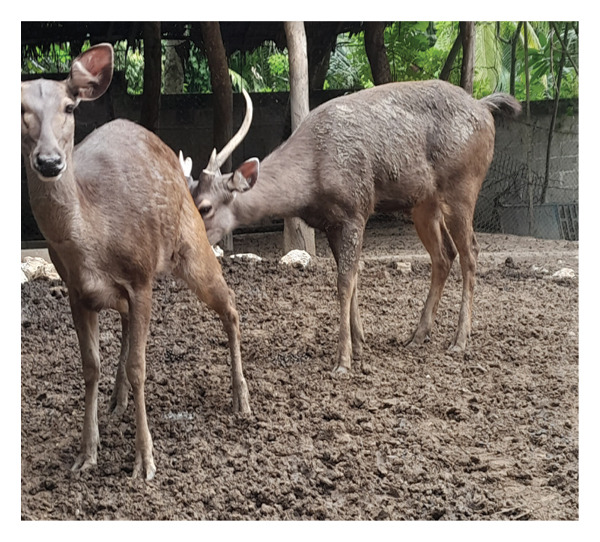
(c)

**Figure:**
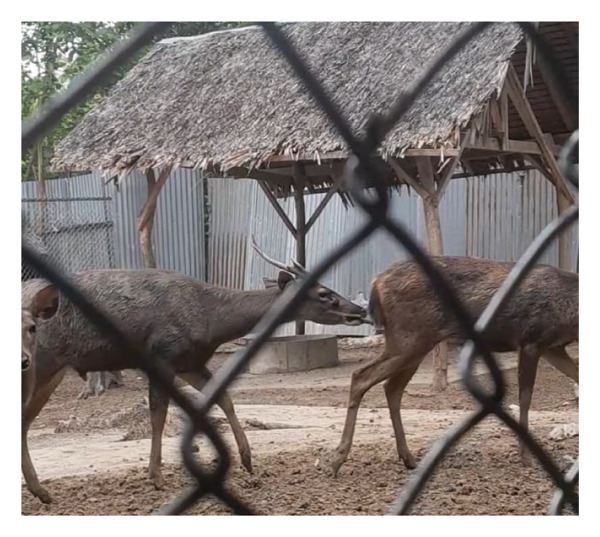
(d)

**Figure:**
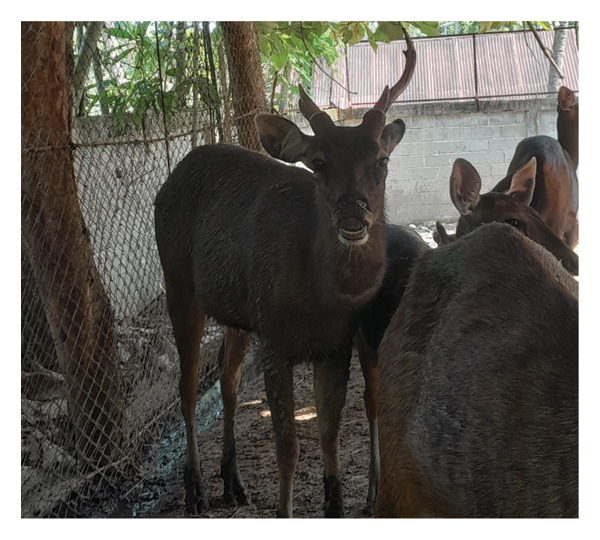
(e)

**Figure:**
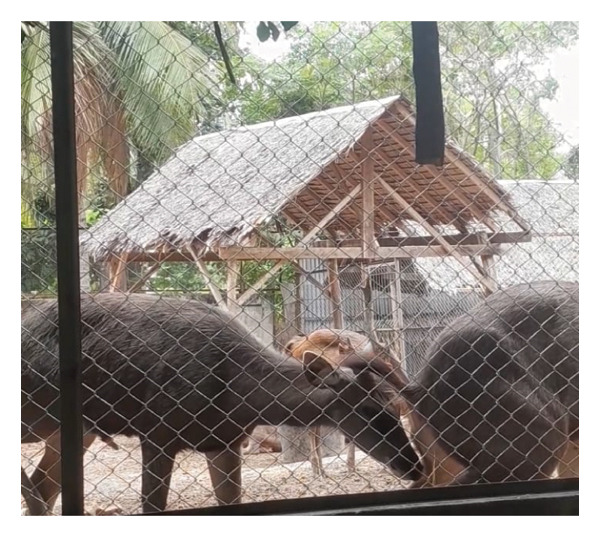
(f)

**Figure:**
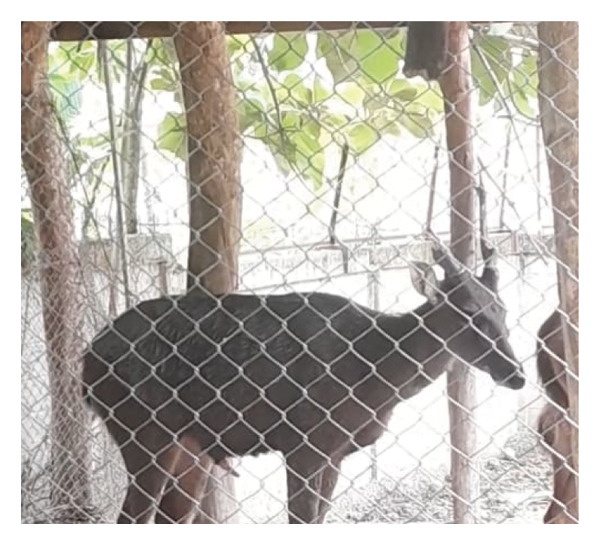
(g)

**Figure:**
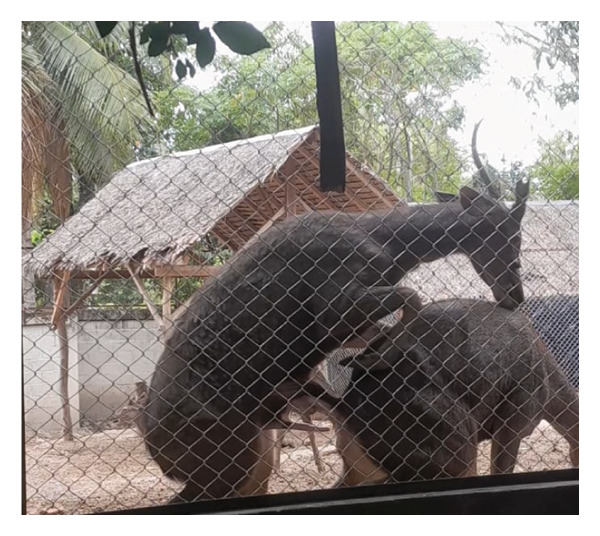
(h)

**Figure:**
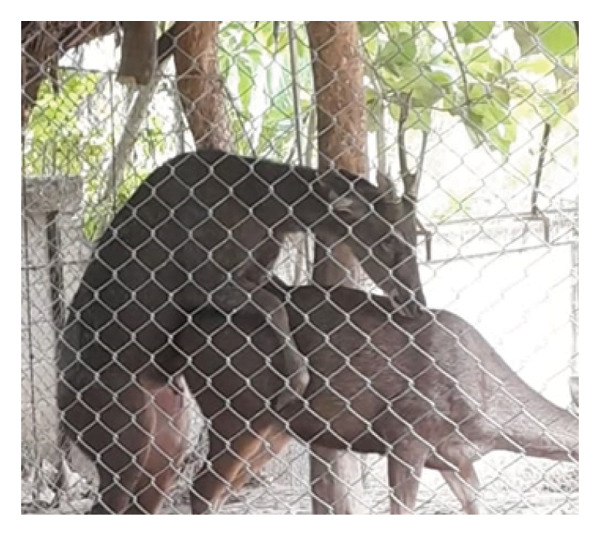
(i)

**Figure:**
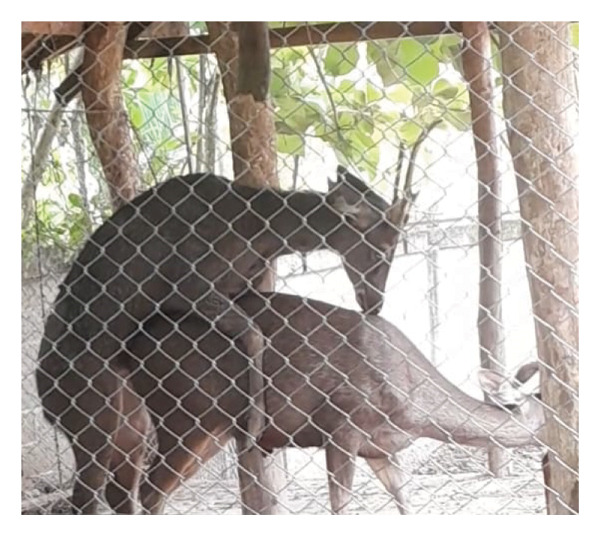
(j)

**Figure:**
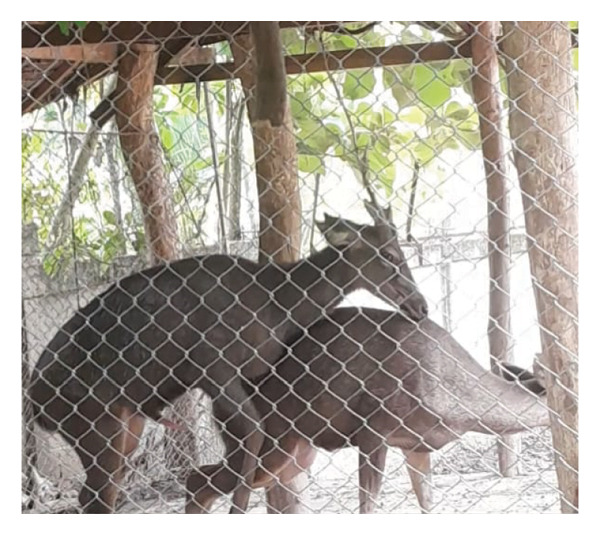
(k)

**Figure:**
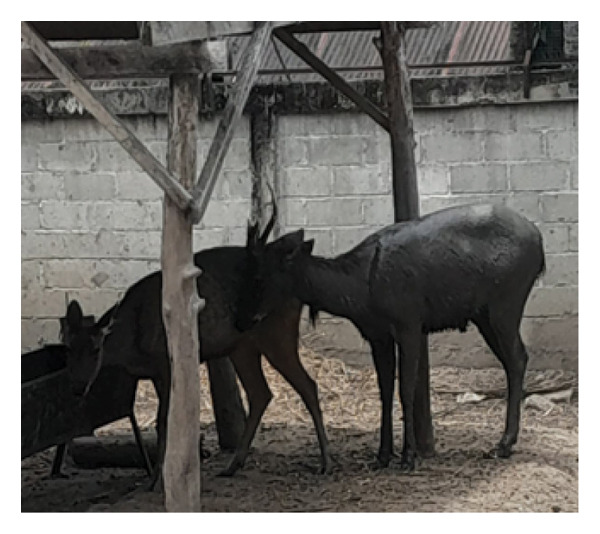
(l)

**Table 4:** Number and duration of sexual behavior in sambar deer stag in different antler stages in Taman Rusa Lamtanjong, Aceh, Indonesia.

Antler stage	Sexual behavior	Specific events	Number of specific events (day)	Total duration (s)	Frequency (%)
Hard antler	Precopulatory behavior	ma	2.13 ± 0.04	20.32 ± 2.57	1.08 ± 0.23
		mf	16.24 ± 3.70	161.15 ± 12.91	8.60 ± 4.52
		as	89.35 ± 13.60	463.36 ± 41.12	47.85 ± 16.76
		al	17.12 ± 4.51	104.14 ± 10.84	9.14 ± 3.21
		fl	17.35 ± 4.60	205.10 ± 12.41	9.14 ± 3.17
		sfu	2.46 ± 0.21	25.38 ± 3.53	1.08 ± 0.41
		er	5.12 ± 1.20	41.27 ± 5.07	2.69 ± 0.56
	Copulatory behavior	mo	7.12 ± 3.41	68.46 ± 18.31	3.76 ± 1.59
		co	4.02 ± 2.35	15.07 ± 3.53	2.15 ± 2.02
	Postcopulatory behavior	ds	5.45 ± 1.40	5.16 ± 0.54	2.69 ± 0.76
		gr	6.22 ± 1.90	28.18 ± 3.47	3.23 ± 1.03

Casting	Precopulatory behavior	as	1.14 ± 0.22	6.52 ± 1.49	0.54 ± 0.45
	Copulatory behavior	n.a	0.00 ± 0.00	0.00 ± 0.00	0.00 ± 0.00
	Postcopulatory behavior	n.a	0.00 ± 0.00	0.00 ± 0.00	0.00 ± 0.00

Velvet	Precopulatory behavior	as	13.34 ± 2.51	42.28 ± 5.16	6.99 ± 5.89
		al	1.01 ± 0.10	3.02 ± 0,21	0.54 ± 0.35
		fl	1.02 ± 0.10	2.05 ± 0.12	0.54 ± 0.85
	Copulatory behavior	n.a	0.00 ± 0.00	0.00 ± 0.00	0.00 ± 0.00
	Postcopulatory behavior	n.a	0.00 ± 0.00	0.00 ± 0.00	0.00 ± 0.00

*Note:* Frequency is calculated by dividing the number of specific sexual behaviors by the total number of sexual behaviors exhibited. fl = flehmen, er = erection, m0 = mounting, co = copulation, ds = dismount, and gr = grooming. n.a = not available or no sexual behavior exhibited.

Abbreviations: al = anogenital licking, as = anogenital sniffing, ma = male approach, mf = male follow, sfu = smelling female’s urine.

Figure 6The correlation between testosterone levels and the number of sexual behaviors in different antler stages in male sambar deer: (a) hard antler, (b) velvet, and (c) casting.

**Figure:**
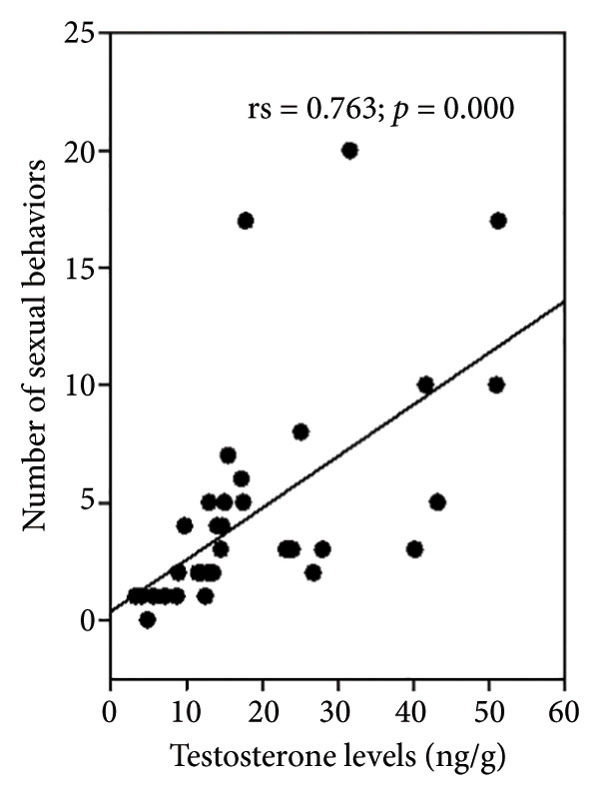
(a)

**Figure:**
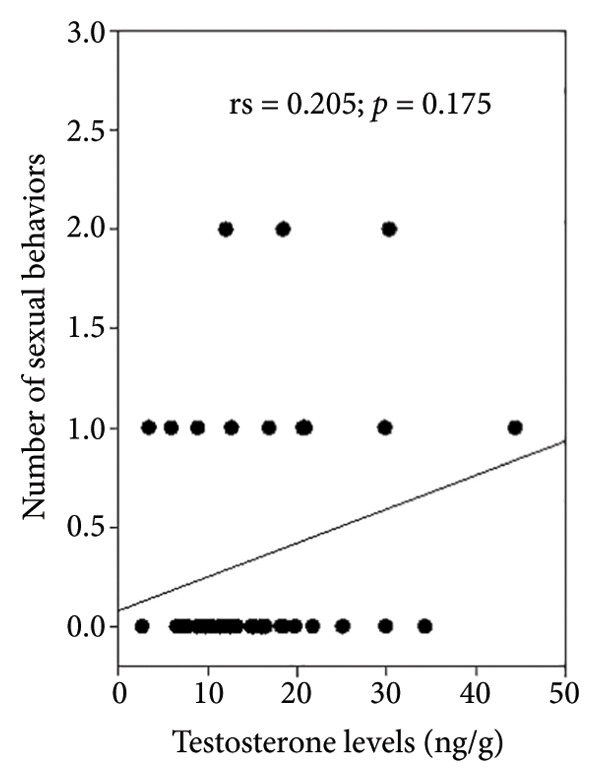
(b)

**Figure:**
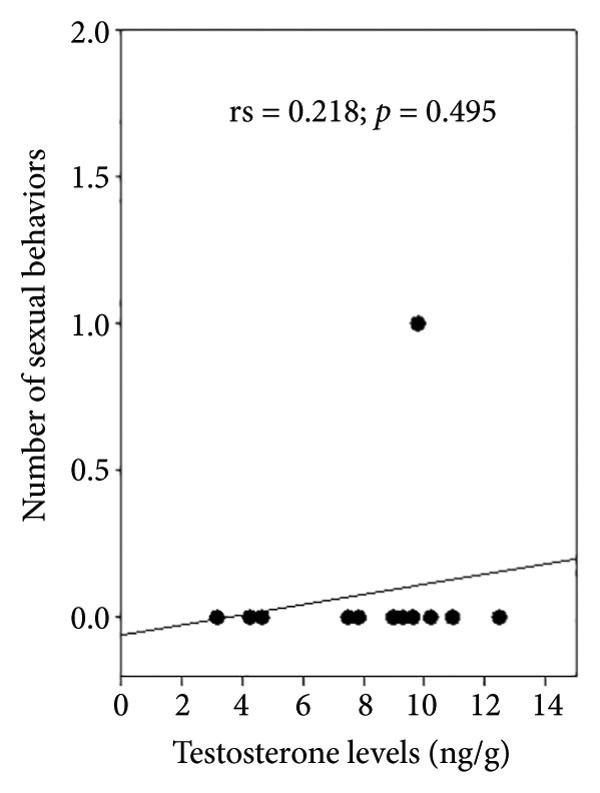
(c)

## 4. Discussion

The findings indicate a significant relationship between testosterone levels, antler stages, and male sexual behaviors of tropical deer. Assay validation confirmed the reliability and physiological relevance of the testosterone assay, supporting the validity of the hormonal data used in this study. Levels of testosterone exhibited variations across different antler stages, with those in the hard and growth (velvet) stages displaying elevated testosterone levels compared to those in the shedding (casting) stage. Additionally, a notable correlation was observed between testosterone levels and male sexual behavior during hard antlers. However, intriguingly, the heightened testosterone levels during the velvet did not appear to correspond with male sexual behavior. While these findings show stage‐specific differences in testosterone levels and their behavioral correlates, they should be interpreted with caution due to the limited sample size and uneven distribution of antler stages, which may reduce statistical power and increase variability in the estimates. Despite these limitations, the consistent directional patterns across individuals provide reasonable confidence in the biological relevance of these trends, while further studies with larger samples and full annual coverage are warranted to confirm these effects.

Testosterone levels during the hard and velvet antler stages are approximately twice as high as compared to the casting stage. These findings align with a similar investigation conducted on sambar deer in the Horton Plains National Park, Sri Lanka [4]. They reported the highest testosterone levels during the hard antler stage (18.56 ± 2.17 ng/g), followed by a gradual increase during velvet (14.18 ± 1.76 ng/g), and a significant decrease during casting, with the lowest testosterone levels (4.58 ± 0.71 ng/g).

The testosterone profile of tropical sambar deer is also similar to temperate deer, as shown in white‐tailed deer. The testosterone level is at its highest levels during mating season and declines afterward until the casting stage, before increasing to the highest level during velvet antler growth to velvet antler shedding [9], while in Iberian red deer, peak serum testosterone level occurs mainly during the rut, at the beginning of the mating season [8]. During the hard antler stage, testosterone plays a pivotal role in coordinating various aspects of the stag sambar deer’s reproductive behavior and physiology. Elevated levels of testosterone stimulate the development and hardening of antlers [4], which are crucial for dominance displays and competition among males for mating opportunities. Moreover, heightened testosterone levels during this stage act as a key regulator of sexual behavior in sambar deer stags. Significantly higher numbers of sexual behaviors (91.40%) were observed during the hard antler stage compared to the velvet and casting stages.

Sambar deer stags primarily exhibited precopulatory behaviors, particularly courtship behaviors, such as anogenital sniffing, anogenital licking, and flehmen (66.13%). These behaviors are critical for scent marking and acquiring olfactory information related to female receptivity and reproductive status [22]. Furthermore, during the hard antler stage, sambar deer stags were observed mounting (3.76%) and successfully copulating (2.15%) with females. Monitoring the sexual behaviors, particularly copulatory behaviors, provides an estimate of the timing and duration of the estrous period [23]. Observing sexual behavior can increase reproductive success by aligning with female receptivity [24]. The higher number of sexual behaviors during the hard antler stage can be used for stag mating management, maximizing mating success under optimal conditions. It is essential to note that fecal testosterone levels reflect circulating hormone activity with a physiological delay. In this study, this aspect was accounted for by considering an estimated 1‐ to 2‐day excretion lag when correlating fecal testosterone concentrations with male sexual behaviors. This estimate is consistent with findings in related cervid species and small ruminants, in which fecal steroid metabolites appear approximately 18–36 h after secretion into circulation [20, 21]. Therefore, the observed correlations between testosterone and mating behaviors should be interpreted as reflecting endocrine activity from the preceding day(s) rather than instantaneous hormonal changes.

The correlation between testosterone levels and male sexual behaviors observed during the hard antler stage underscores the critical role of androgens in regulating reproductive activity in deer. Several factors may account for these results. During the hard antler stage, testosterone levels tend to reach their peak, coinciding with heightened sexual activity among male deer. This surge in testosterone is integral to the development and hardening of antlers, which are pivotal for dominance displays and mating competition among males [25]. Consequently, the elevated testosterone levels during this stage could fuel an increase in male sexual behaviors as they actively vie for mating partners.

Previous studies across various cervid species have also demonstrated a positive association between testosterone levels and dominance rank, as well as male sexual behaviors [26, 27]. Additionally, in male Iberian red deer, testosterone may play a crucial role in sperm production, with peak semen quality observed when testosterone levels are highest [28]. As the hard antler stage concludes, testosterone levels decrease, leading to antler casting. This abrupt decline in testosterone levels during the casting stage likely contributes to the reduced number of sexual behaviors observed in the sambar deer stag. A previous study also reported that the antler cycle of the deer stag appears to influence sexual behavior [29]. Furthermore, a previous study reported an increase in cortisol levels during the casting stage, which could potentially suppress testosterone production [12]. These findings suggest the close association between testosterone levels and male sexual behaviors, particularly during the hard antler stage when mating opportunities are abundant, and competition among males is most intense.

Although testosterone levels remained high during the velvet stage, the frequency of male sexual behaviors was markedly lower compared to that observed in the hard antler stage. This discrepancy suggests that testosterone may play additional roles beyond male competition or mating during the velvet stage [30]. One potential explanation for this finding is that testosterone serves multiple functions throughout the deer reproductive cycle, including the velvet stage. While testosterone is traditionally associated with promoting aggressive behaviors and mating activities during the hard antler stage [31], its role during the velvet stage may be more nuanced. During this stage, male deer are primarily focused on antler growth and development, as the velvet serves as a protective covering for the developing antlers [4]. Therefore, testosterone may primarily facilitate physiological processes related to antler growth and maintenance rather than driving aggressive or mating behaviors [32].

Furthermore, the sustained elevation of testosterone during the velvet stage could also be attributed to its involvement in maintaining overall reproductive readiness. Testosterone plays a crucial role in regulating various physiological functions beyond mating behaviors, including maintaining muscle mass, bone density, and overall vitality [33]. Therefore, the sustained high levels of testosterone during the velvet stage may indicate its role in supporting overall reproductive fitness and readiness for future mating opportunities [34]. Previous studies have demonstrated that testosterone plays a crucial role in stimulating and sustaining antler growth [8], modulating aggression and dominance behaviors [29], and influencing systemic trade‐offs, such as immune function and energy balance [35, 36]. These broader functions may explain the persistence of high testosterone levels outside the peak mating period. Future studies integrating hormonal, behavioral, and physiological markers are needed to clarify the multifunctional role of testosterone in tropical cervids.

Our study was conducted over a limited period and did not capture a complete antler cycle in all individuals, which makes it difficult to determine whether the observed hormonal and behavioral patterns aligned with the regional peak breeding season. Nevertheless, the findings clearly demonstrate a strong association between testosterone, antler stage, and sexual behavior, providing important insights into the reproductive biology of a tropical cervid species with more flexible breeding strategies. Although estrus observations were not systematically recorded, this focus on male endocrinology establishes a foundation for future work. Integrating both male and female reproductive parameters in longitudinal studies that span full annual cycles will be particularly valuable to clarify seasonal influences and the interplay between male physiology, female receptivity, and social behavior in sambar deer. Importantly, despite these limitations, this study represents a significant first step toward understanding testosterone–behavior dynamics in tropical cervids, offering a basis for future research with larger sample sizes and more comprehensive monitoring.

## 5. Conclusion

Levels of testosterone in male sambar deer fluctuated between antler stages, peaking during the hard antler stage, falling during casting, and then increasing again during the velvet stage. While there was a considerable positive link between testosterone levels and sexual behaviors during the hard antler stage, no significant correlation was detected during the velvet or casting stages, implying that testosterone levels have different roles at each antler stage.

## Disclosure

All authors have read and agreed to the published version of the manuscript.

## Conflicts of Interest

The authors declare no conflicts of interest.

## Author Contributions

Each author contributes equally to this study. Conceptualization: Gholib Gholib and Muslim Akmal; Methodology: Gholib Gholib and Sri Wahyuni; Data curation: Dara Cut Rinjani and Taufiq Purna Nugraha; Data analysis: Gholib Gholib and Dara Cut Rinjani; Resources: Gholib Gholib and Muslim Akmal; Writing–original draft: Gholib Gholib and Dara Cut Rinjani; Writing–review and editing: Gholib Gholib, Muslim Akmal, Sri Wahyuni, and Taufiq Purna Nugraha.

## Funding

The authors express their gratitude to the Universitas Syiah Kuala for funding this study through the H‐Index Research 2023 grant (Grant No. 124/UN11.2.1/PT.01.03/PNBP/2023).

## Data Availability

The data supporting the findings of this study are available from the corresponding author.
